# Manufacturing technology of banana‐assorted breads: The fermentative characteristics affected by different banana cultivars

**DOI:** 10.1002/fsn3.1539

**Published:** 2020-04-30

**Authors:** Li‐Yun Lin, Chiung Chi Peng, Kuan‐Chou Chen, Hui‐Er Wang, Chun‐Shen Wang, Kun Hung Shen, Robert Y. Peng

**Affiliations:** ^1^ Department of Food and Applied Technology Hungkuang University Taichung City Taiwan; ^2^ Graduate Institute of Clinical Medicine College of Medicine Taipei Medical University Taipei Taiwan; ^3^ Department of Urology Taipei Medical University Shuang‐Ho Hospital Taipei Taiwan; ^4^ Division of Urology Department of Surgery Chi‐Mei Medical Center Yung Kang City Taiwan

**Keywords:** banana breads processing, fermentative characteristics, hedonic scoring, *Musa sapientum* and *M. formosana*, pectin, soluble sugars, textural profile analysis

## Abstract

Taiwan produces large quantities of bananas in the southern area. Recently, due to the export quantity has been greatly reduced, in order to efficiently maintain the banana agriculture and economy, the development of alternate uses of bananas has become urgently in need. Bananas contain a fair amount of nutrients with low glycemic index. Currently, as the bread consumption is increasing, we tried to manufacture banana‐assorted breads. The desiccated powders of *Musa sapientum* var TC2‐425 Linn [(genomically, called as *Musa* (AAA) (MA)] and *Musa basjoo* “Nam Wa” (MB) were separately incorporated at 15%, 20%, and 25% (denoted as MA15‐MA25 and MB15‐MB25). Results indicated that MA exhibited higher contents of moisture, ash, crude protein, and lutein, while with lower crude fat, crude fibers, carbohydrate, sodium, total soluble sugars, and pectin. The contents of taste compounds (name, samples in decreasing order) were as follows: 5′‐CMP (MB25, MB20); 5′‐GMP (MA25, MA20); 5′‐AMP (MB25, MA15); 5′‐XMP (MA25, MA20); 5′‐IMP (MA25, MB20, MB25); and 5′‐UMP (MA20, MA25, MB20). Hedonic scoring (HS) indicated MA15, MA20, MB15, and MB20 were more acceptable. Textural profile analysis (TPA; for 0–6 days, only 0–4 days are shown) revealed that “flavor,” “mouthfeel,” “hardness,” “gumminess,” and “chewiness” were the determinant key roles. Conclusively, due to different chemical constituent of banana, different recipes must be considered. The bread acceptability is affected by the fermentative profile which in turn is governed by the contents of soluble sugars, pectin, taste compounds, and the overall activity of yeast cells.

## Graphical abstract

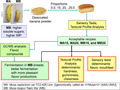

## 1 INTRODUCTION

After 1967, Taiwan began producing large quantities of bananas in the southern Taiwan area, with an area under cultivation of over 50,000 hectares, exporting over 400,000 tons per year and thereby accruing significant foreign currency. According to a report in 2019, the productive quantity reached 295,000 metric tones = 0.295 million metric tones/year (https://www.worldblaze.in/banana‐producing‐countries‐in‐the‐world/, 2019; https://en.wikipedia.org/wiki/Banana, 2017; https://www.statista.com/statistics/321795/taiwan‐banana‐production/, 2005–2016).

Once Taiwan bananas had been highly evaluated in Japan. As Taiwan bananas taste much sweeter and more adhesive, accordingly they gain higher wharf side prices than the product from other areas (Yoshiyuki, 2006).

Bananas contain a fair amount of fiber, as well as several antioxidants. The proximate composition of desiccated (hot air dried) banana flours has been reported to contain moisture 14.31 ± 0.6%, protein 3.19 ± 0.08%, fat 0.50 ± 0.05%, ash 1.20 ± 0.09%, fiber 4.2 ± 0.1%, and carbohydrate 80.80 ± 0.05%; and calcium 32 ± 0.2 mg, phosphorus 93 ± 0.4 mg, iron 2.6 ± 0.1 mg, and zinc 0.18 ± 0.1 mg (Asif‐Ul‐Alam, Islam, Hoque, & Monalisa, 2014). In addition, it contains abundant vitamin A, vitamin E, and mineral ions (Vaughan, Geissler, Nicholson, Dowle, & Rice, 2009). Similar results have been reported by Borah (2018).

**Figure 1:**
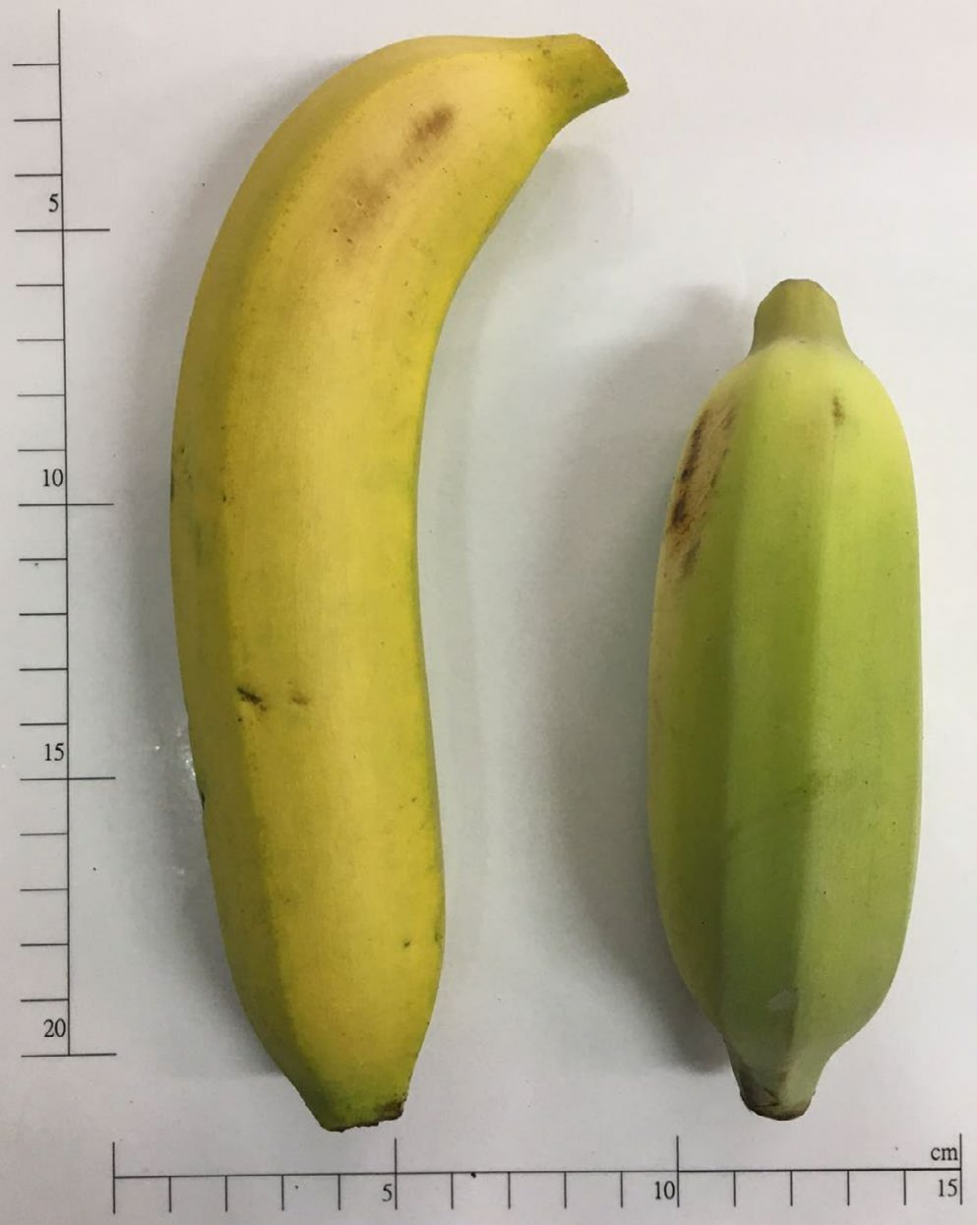
Size comparison between banana fruits from *Musa sapientum* var TC2‐425 Linn [(genomically, called as *Musa* (AAA) (MA)] and *Musa basjoo* “Nam Wa” (MB)

Bananas are beneficial to cardiovascular system for its good source of potassium, which could help maintain normal blood pressure and heart function, preventing hypertension and protecting against atherosclerosis. In addition, bananas are rich in antioxidant components including polyphenolics, isoflavonoids, and dopamines (Someya, Yoshiki, & Okubo, 2002). Its high dietary fiber content is helpful to peristalsis and digestion (Aurore, Parfait, & Hrasmane, 2008). Nutritionally, bananas are low in the glycemic index. According to the International GI Database, fully ripened banana has a glycemic index of 51. Although bananas are a very low‐fat food (less than 4% of their calories come from fat), they contain small amounts of sterols like sitosterol, campesterol, and stigmasterol which can block the absorption of dietary cholesterol, decrease low‐density lipoprotein cholesterol, and moderate the glucose response (Baker, 1994; Reiser, 1987). Occasionally, the competition from other banana‐producing countries and the overproduction of bananas occur in Taiwan, which may cause both economical and political instability in Taiwan, and the development of alternate uses of bananas and its related manufacturing process have become urgently in need. Currently, as life style is changing, the yearly bread consumption is increasing due to the reasons of easy handling and time‐saving. For the past decades, banana spreads has long been a delicious toast spread. Considering the nutritional value of banana and huge productivity in Taiwan, we tried to incorporate the desiccated banana fruit powder into the toast recipes. For comparison, fruits from *Musa sapientum* var TC2‐425 Linn (genomically, called as *Musa* (AAA)) (MA) [so far it might have been wrongly identified as *Musa formosana* (Wall.)] and *Musa basjoo* “Nam Wa” (MB) were separately used in the recipes of different toasts. Their size comparison is shown in Figure 1.

The former (MA; a triploid banana) is starchier, and the latter (MB; a diploid banana) has fresh of more yellow images.

## 2 MATERIALS AND METHODS

### 2.1 Chemicals and reagents

Super high gluten flour was gifted by Chia‐Fa flour Co. (Taichung, Taiwan). *Musa sapientum* var TC2‐425 Linn (genomically, should be called as *Musa* (AAA)) (MA) [so far this might have been wrongly identified as *Musa formosana* (Wall.)] and *Musa basjoo* “Nam Wa” (MB; Ripeness color score, C7, Appendix Figure A1) were purchased from Nan‐Tou County, (Taiwan). Other ingredients were supplied by the local agent. All other reagents unless otherwise mentioned were provided by Katayama Chemical Industries Co. Ltd.

### 2.2 Preparation of banana powder

Banana fruits of MA and MB were rinsed and cut at the head and end, cut into thin slices with thickness of 0.2–0.3 cm, subjected to hot air blowing dryer at 50°C until dry, and frozen stored. The desiccated banana slices were freshly pulverized with a grinder before use. The powder was sieved, and those particles having size below 0.4 mm were collected for use (the banana powder is denoted hereafter as “DBP” here after).

### 2.3 Recipes for bread making

The basic constant ingredients were (expressed in proportion) as follows: milk 4, yeast 1, salt 1.5, egg 8, and shortening 10. The varying ingredients were DBP, either MA or MB, in proportions of 0.0, 15.0, 20.0, and 25.0; strong flour, in 100, 85, 80, and 75; and water, in 52, 47.2, 45.6, and 44, respectively (Table 1).

**Table 1:** Recipes for making toast breads

Ingredient	Recipes			
Banana powder^a^	0	15	20	25
Strong flour	100	85	80	75
Milk powder	4	4	4	4
Yeast	1	1	1	1
Salt	1.5	1.5	1.5	1.5
Egg	8	8	8	8
Shortening	10	10	10	10
Water	52	47.2	45.6	44
Total	186.5	181.7	180.1	178.5

^a^ The desiccated banana powders used included either *Musa sapientum* var TC2‐425 Linn [(genomically, called as *Musa* (AAA) (MA)] or *Musa basjoo* “Nam Wa” (MB).

### 2.4 Bread making

The experimental baking was a small‐scale straight‐dough baking test according to the Berlin Institute (Jakubczyk & Haber, 1983). For primary fermentation, the dough was fermented at 28°C and 75% RH for 75 min (with 1 min transfixion after 30 min) in the fermentation cabinet, the intermediate fermentation, at 28°C, RH 75%, for 15 min. The final fermentation was conducted at 38°C, RH 85%, for 50 min. Then, the loaves were baked in an oven (live steam was injected immediately after the loaves were placed in the oven) at 180°C (upper) and 200°C (lower) for 35 min and packed after cooled. The baking tests were carried out in triplicates.

### 2.5 Preparation of banana‐assorted toast powders

The finished toasts were stored at 25°C for 2, 4, and 6 days, respectively, blended, ground, and screened through #60 mesh. The desiccated banana‐assorted toast powder (#60) was transferred into a plastic seal bag and sealed tightly (assigned as DBB) for analysis.

### 2.6 Proximate compositional analysis

The proximate compositional analysis of all related stuffs was carried out according to the protocol described in Manual of Food Analytical Protocols (1990). Method of AOAC 2011.25 was followed to analyze the content of dietary fibers.

### 2.7 Determination of reducing sugars

The dinitrosalicylic acid (DNS) protocol recommended by Miller (1959), and Hughes and Lindsay (1985) was followed. The final solution was measured spectrophotometrically at 540 nm using Hitachi U‐2001 spectrophotometer. A calibration curve was established using authentic glucose (Sigma‐Aldrich), from which the amount of reducing sugar was calculated.

### 2.8 Analyses of the taste components

#### 2.8.1 Determination of nucleotide content

According to the protocol described by Taylor, Hershey, Levine, Coy, & Olivelle (1981), to 5 g powder of plain toasts, *M*. *sapientum*‐, and *M*. *basjoo*‐assorted toasts, 25 ml deionized water was added. The remaining procedures were conducted as instructed. Ten microlitre aliquot of final concentrated extracts was subjected to Hitachi High Performance Liquid Chromatography (HPLC; L‐2130, Japan) analysis at 254 nm, using an Ascentis C18 column (ℓ × i.d. = 250 mm × 4.6 mm, particle size, 5 μm) equipped with a Hitachi L‐4000UV detector connected to the Hitachi D‐2500 Chromato‐Integrator. The mobile phase used was 0.5 M potassium dihydrogen phosphate (pH 4.0). The flow rate was set at 0.4 ml/min. The calibration curves were established using different authentic nucleotide samples, from which each nucleotide of interest was determined.

#### 2.8.2 Determination of soluble sugars

Method of Ajlouni, Beelman, Thompson, & Mau, (1995) was followed to determine the soluble sugar content. Different authentic sugars were used to establish the calibration curves, from which the amount of each soluble sugar was calculated. The operation conditions for HPLC were as follows: Shimadzu LC‐10AT *VP* equipped with a Pinnacle II Amino column (ℓ × i.d. = 250 mm × 4.6 mm, particle size, 5 μm), a Shimadzu RID‐10A detector, and a software SISC 32 Chinese version 2.1 for data processing. The mobile phase consisted of acetonitrile: water = 70:30 (v/v) at a flow rate 1 ml/min.

### 2.9 Determination of pectin content

The pectin content was determined according Kulkarni and Vijayanand (2010). The final desiccated pectin was weighed (*W*_p_ g). The yield of pectin was calculated as follows:

(1) $$\text{Pectin}\;\left(\%,w/w\right)=\left(W_{p}/W_{b}\right)\times 100$$

### 2.10 Determination of lutein content

The content of lutein in DBP was determined according to Hart and Scott (1995). The final two successive extracts were combined and made to 100 ml with BHA (in acetone). An aliquot of 20 ml was measured and filtered through 0.2‐μm syringe filter. The optical density was measured at 445 nm. The content of lutein was calculated according to Equation 2.

(2) $$\text{Lutein}\;\left(\mu g/\text{ml}\right)=[{\text{OD}}_{445\text{nm}}\times 5680000/W_{\text{sample}}\left(\text{mg}\right)]/122688$$

### 2.11 Preparation and analysis of volatiles

#### 2.11.1 Steam distillation

The essential oil was obtained from the desiccated bread powder (500 g) by steam distillation. The essential oils obtained were weighed, and the yields were calculated. The products were stored at 20°C for GC analysis.

#### 2.11.2 GC/MS analysis

A gas chromatography GC HP 6890 attached to a HP5973MSD detector and a capillary column DB‐1 (*h*) 60 m; i.d. = 0.25 mm; and membrane thickness (0.25 μm) was used. Nitrogen was used as the carrier gas and operated at a flow rate of 1 ml/min. The temperature at the injection port was set to 250°C. The ionization potential used was 70 eV, where the temperature of the ion source was held at 230°C. Initially, the temperature was set at 40°C for 10 min, then programmed at 2°C/min up to 240°C, and held at this temperature for 30 min. The flux ratio was set at 80:1.

#### 2.11.3 Quantification and identification of volatile constituents

Aliquots (1.0 μl) of the essential oils were, respectively, measured with a GC microsyringe and analyzed with GC and GC/MS. Quantification of each constituent was calculated from the integrated diagrams. A reference mixture of *n*‐paraffins (C_5_–C_25_) was used to calculate the retention indices (RI) under the same conditions. The RI obtained from GC are determined by analogy with Kovats indices for the following reference compounds: benzene, naphthalene, phenanthrene, chrysene, and picene (Kovats, 1958):

(3) $$R\text{.I}.=\left[\left(\log\left(t_{r}^{\prime }\right)x-\log\left(t_{r}^{\prime }\right)n/\left(\log\left(t_{r}^{\prime }\right)n+1\right)-\log\left(t_{r}^{\prime }\right)n\right)\right]+100n$$

where $t_{r}^{\prime }=\left(t_{r}-t_{m}\right)$ is the calibrated and corrected retention time for each chemical. *n* is the carbon number of n‐alkanes. *t*_m_ is the retention of methane in the column. *t*_r_ is the retention time of chemicals in the column. *x* is the unknown chemical.

By referring to the documented data, each exact constituent was deduced. Alternatively, by comparing the GC/MSD spectra, each component was qualitatively matched out and confirmed by GC/MS. The determination for volatile structures was based on the TNO (1996), the Browse‐Wiley Computerized Data Base, the NSB Computerized Data Base, and the cited standard spectra of standard chemicals.

### 2.12 Texture profile analysis (TPA)

Textural properties of bread crumbs were tested by following the TPA protocol as reported (Steffe, 1996; Wang, Rosell, & Benedito de Barber, 2002). Twelve replicates of bread crumb sample were analyzed. The parameters recorded were hardness, springiness, cohesiveness, gumminess, chewiness, and resilience. The procedure was repeated for each determination. Real‐time data acquisition was accomplished by following the TAXT2’s User Guide (Anonymous, 1977). The software was used to calculate hardness (kg), cohesiveness, chewiness, gumminess, and adhesiveness values of the bread samples (Bourne, 1982). The TPA values reported are the averages of 3 different determinations.

### 2.13 Time‐dependent image change of the bread texture

The finished sample white toasts and banana‐assorted toasts were stored at 25°C, and the photograph of the texture was taken at day 0 and day 4, respectively, with Canon (type: PowerShot S100 12.1 MP Digital Camera with 5× Wide‐Angle Optical Image Stabilized Zoom, Black).

### 2.14 Storage tests

The assorted toast breads were stored at 25°C for 6 days, and the textural profile analysis was carried out. The parameters included hardness, springiness, cohesiveness, gumminess, chewiness, and resilience.

### 2.15 Estimation of specific volume of loaves

Loaf weight (g), loaf height (cm), loaf volume (cm^3^), and loaf specific volume (cm^3^/g) of all bread samples were examined according to the methods of AACC (2000). After baked, the loaf volume was measured by the rapeseed displacement method (Greene & Bovell‐Benjamin, 2004). The specific volume was calculated according to Equation 4.

(4) $$\rho \left({\text{cm}}^{3}/g\right)=V_{\text{loaf}}/W_{\text{loaf}}$$

where *ρ* is the specific volume in cm^3^/g, *V*_loaf_ is the volume of bread sample in cm^3^, and *W*_loaf_ is the weight of loaf in *g*.

### 2.16 The analysis of color differences

The color difference in desiccated SPM peel powder, toast breads, and the SPM peel‐assorted toast breads was analyzed with a colorimeter, Σ80 Color Measuring System (Nippon Denshoku Inc., Co., LTD), according to the method cited by Malik, Nayik, and Dar (2015). The parameters L (brightness parameter), a (red‐green), and b (yellow‐blue) were measured and calibrated against the standard color plate with parameters *Y* = 86.76, *X* = 81.73, and *Z* = 92.56. Readings were displayed as L* (black to white), a*(redness to greenness), and b* (blueness to yellowness) color parameters according to CIELAB system of color measurement (Malik et al., 2015). The samples were measured in triplicates. The combined characteristic color was expressed as whiteness index (Rhim, Wu, Weller, & Schnepf, 1999).

where

(5) $$\text{WI}={\left[\left(100-L^{*2}\right)+a^{*2}+b^{*2}\right]}^{1/2}$$

### 2.17 Sensory evaluation by hedonic scoring system

This study was approved by the Human Ethics Committee at the HKU and followed strict norms of the resolution of the Ministry of Health and Common Welfare (Taiwan). The sensory evaluation of breads was conducted and criticized by 50 untrained panelists recruited from the Department of Food And Applied Technology, Hungkuang University (HKU) community. For rating, five attributes, including appearance, color, flavor, and mouthfeel, and the overall acceptability were referred according to the instructions given by the International Standard Organization (1982). This study was approved by the Human Ethics Committee at the HKU and followed strict norms of the resolution of the Ministry of Health and Common Welfare (Taiwan).

The sensory characteristics, that is, crust color, crumb color, oral texture, manual texture, aroma, taste, and overall acceptability, were determined on the basis of seven‐point hedonic scale (representing 1 = disliked very much, 2 = disliked moderately, 3 = dislike slightly, 4 = neither liked nor disliked, 5 = liked slightly, 6 = liked moderately, and 7 = liked very much) Land and Shepherd (1984).

### 2.18 Variation of the hydration status

The moisture content of breads was measured on days 0, 2, 4, and 6. The percent water content variation was calculated according to Equation 6.

(6) $$\%\;\text{moisture}\;\text{charge}=[\left(W_{t}-W_{0}\right)/W_{0}]\times 100$$

where *W*_t_ = the moisture content on day 2 and/or day 4; *W*_0_ = the initial moisture content on day 0.

### 2.19 Statistical analysis

Triplicate data obtained in the same group were analyzed by Student's *t* test with computer statistical software SPSS 10.0 (SPSS). The software from Statistical Analysis System for one‐way analysis of variance (one‐way ANOVA) was used to analyze the variances. Duncan's multiple range tests were used to test their significances of difference between paired means. Significance of difference was judged by a confidence level of *p* < .05.

## 3 RESULTS AND DISCUSSION

### 3.1 Proximate composition

Both MA and MB exhibited larger caloric content than white toasts, ranging within 340 to 360 kcal/100 g (Table 2). MA exhibited higher ash content (4.8 ± 0.1 g/100 g) than MB (3.0 ± 0.2 g/100 g), while both were far higher than that of white toast (1.5 ± 0.1 g/100 g; Table 2). The crude protein content was highest for the white toast compared to 4.6 ± 0.1 g/100 g and 1.5 ± 0.1 g/100 g, respectively, for MA and MB, and similar result was observed for the crude fat content (Table 2). MA showed lower content of crude fiber and carbohydrate content than MB (for crude fiber, 5.4 ± 0.1 g/100 g vs. 6.4 ± 1.1 g/100 g and for carbohydrate content, 74.6 ± 1.2 g/100 g vs. 80.4 ± 0.6 g/100 g; Table 2), compared to 51.3 ± 3.5 g/100 g of carbohydrate content in the white toasts (Table 2). And as expected from the recipes, the white toasts exhibited the highest content of sodium (691.2 ± 12.6 mg/100 g). Worth noting, all recipes contained no any trace of saturated fatty acids and trans fatty acids (Table 2), implicating the banana‐assorted toasts to be safe and beneficial to health. On the other hand, Arnarson's report (Arnarson, 2014) was different in some parameters from our data. Such a great discrepancy might be caused by (a) different cultivars of bananas, and (b) different maturation stages, (c) different climate, and (d) different soil composition.

**Table 2:** Proximate compositional analysis for the two banana cultivars^1^

Items	Sample		
	MA	MB	Control
Calories (kcl/100 g)	340.2 ± 0.1^b^	360.8 ± 0.1^a^	332.9 ± 1.2^b^
Moisture (g/100 g)	12.6 ± 0.1^d^	10.1 ± 0.9^e^	27.9 ± 2.1^b^
Ash (g/100 g)	4.8 ± 0.1^a^	3.0 ± 0.2^b^	1.5 ± 0.1^e^
Crude protein (g/100 g)	4.6 ± 0.1^d^	1.5 ± 0.1^e^	9.3 ± 0.8^a^
Crude fat (g/100 g)	2.6 ± 0.1^f^	3.7 ± 0.1^e^	10.09 ± 1.1^a^
Crude fiber (%)	5.4 ± 0.1^b^	6.4 ± 1.1^a^	—
Saturate fatty acids (g/100 g)	0.0 ± 0.0	0.0 ± 0.0	0.0 ± 0.0
Trans fatty acids (g/100 g)	0.0 ± 0.0	0.0 ± 0.0	0.0 ± 0.0
Carbohydrate (g/100 g)	74.6 ± 1.2^b^	80.4 ± 0.6^a^	51.3 ± 3.5^c^
Sodium (mg/100 g)	119.2 ± 7.2^i^	148.9 ± 2.0^g^	691.2 ± 12.6^d^

Data expressed in mean ± *SD*. The superscripts in lower case in each column indicate significant composition difference between banana cultivars.

^1^ *Musa sapientum* var TC2‐425 Linn [(genomically, called as *Musa* (AAA) (MA)] and *Musa basjoo* “Nam Wa” (MB).

### 3.2 Content of pectin

Banana contains two main types of fiber, pectin, and resistant starch, which can be used as markers for denoting the ripening of bananas. MA consisted of lower pectin (4.2 ± 0.2% vs. 5.3 ± 0.3%; Table 3). Baker (1997) indicated that the pectin range of bananas given in Campbell and Palmer was 0.59%–1.28%. Kawabata and Sawayama (1974) examined bananas from several countries and found levels of calcium pectate ranging from 0.55% to 0.68% (averaged 0.63%), which was in good agreement with Medina (1968). Wade, Kavanaugh, Hockley, and Brady (1992) later reported total uronic acid levels of bananas decreased from 1.02% to 0.44% during 8‐day ripening. Obviously, our data seemed to be too high compared to these cited (Kawabata & Sawayama, 1974; Wade et al., 1992). Suggestively, such a discrepancy could be caused by the contamination with some impurities, cultivars variation, and degree of ripening. Worth noting, high pectin content could stimulate pectinase.

**Table 3:** Content of soluble sugars, pectin, and lutein in the two banana cultivars and related products^1^

Sample	Soluble sugar (g/100 g)			Pectin (%)	Lutein (μg/g)
	Fructose	Glucose	Sucrose		
MA	^A^12.4 ± 0.2^b^	^A^13.3 ± 0.7^b^	^A^12.3 ± 0.4^a^	4.2 ± 0.2^b^	724.4 ± 38.8^a^
MB	^B^14.6 ± 0.1^a^	^A^34.9 ± 0.3^a^	^C^5.5 ± 0.1^b^	5.3 ± 0.3^a^	266.8 ± 20.0^b^
White toast (control)	^A^8.0 ± 0.2^c^	^B^6.1 ± 0.4^f^	^C^2.8 ± 0.1^f^	—	—
MA toast (%)					
15	^B^6.5 ± 0.4^f^	^B^6.45 ± 0.35^f^	^A^10.0 ± 0.3^b^	—	—
20	^C^7.5 ± 0.1^e^	^C^7.50 ± 0.14^e^	^A^12.3 ± 0.1^a^	—	—
25	^B^8.1 ± 0.5^d^	^B^8.05 ± 0.49^d^	^A^13.2 ± 0.1^a^	—	—
MB toast (%)					
15	^B^6.7 ± 0.1^f^	^B^6.65 ± 0.07^f^	^B^6.4 ± 0.1^d^	—	—
20	^AB^8.7 ± 0.5^c^	^AB^8.65 ± 0.49^c^	^B^8.5 ± 0.4^c^	—	—
25	^B^9.3 ± 0.3b^c^	^B^9.30 ± 0.28b^c^	^C^8.2 ± 0.2^c^	—	—

Note

Data are expressed in mean ± *SD* (*n* = 3). Different superscripts in upper case on the left corner in the same row indicate significantly difference (*p* < .05). Different superscripts in lower case on the right corner in the same column indicate significantly different (*p* < .05).

^1^ *Musa sapientum* var TC2‐425 Linn [(genomically, called as *Musa* (AAA) (MA)] and *Musa basjoo* “Nam Wa” (MB).

### 3.3 Content of lutein

Astonishingly, MS contained rather high content of lutein (MA vs. MB: 724.4 ± 38.8 vs. 266.8 ± 20.0; Table 3). Lutein has long been associated with vision protection via reducing oxidative stresses in the eye and by lowering chronic inflammation that can contribute to cataracts and age‐related macular degeneration (Leermakers et al., 2016).

### 3.4 Content of taste compounds

#### 3.4.1 Content of soluble sugars

MA and MB, respectively, contained soluble sugars (in g/100 g, in order of fructose, glucose, and sucrose): 12.4 ± 0.2, 13.3 ± 0.7, and 12.3 ± 0.4 in MS vs. 14.6 ± 0.1, 34.9 ± 0.3, and 5.5 ± 0.1 in MB (*p* < .05), comparing with 8.0 ± 0.2, 6.1 ± 0.4, and 2.8 ± 0.1 of the white toasts (*p* < .05; Table 3). The content of fructose, glucose, and sucrose in MA and MB increased with the increased percentage of banana powder, except the sucrose content in MB25, implicating the abundant and sufficient presence of certain sucrose‐hydrolyzing activators in MB25 (Table 3).

#### 3.4.2 The content of taste nucleotides

The 5′‐nucleotides are beneficial as flavor enhancers in soups, gravies, bouillons, and other foods at very low levels and can be used to replace beef extract (Kuninaka, 1969).

MA15 and MA20 were all rich in 5′‐UMP, 5′‐XMP, and 5′‐GMP, while MS25 was rich in 5′‐UMP, 5′‐IMP, and 5′‐XMP (Table 4). As contrast, MB15 was rich in 5′‐IMP, and next 5′‐UMP, and 5′‐XMP, 5′‐AMP, and 5′‐GMP in comparable amounts (35.1–35.7 μg/g dry matter). MB20 contained (in μg/g dry matter) 5′‐UMP, 126.6 ± 0.1; 5′‐IMP, 96.4 ± 0.1; and 5′‐GMP, 38.6 ± 0.2 (Table 4), and interestingly, MB25 contained (in μg/g dry matter) 5′‐UMP, 103.7 ± 0.1; 5′‐CMP, 88.1 ± 0.2; and 5′‐IMP, 65.4 ± 0.1 (Table 4). Apparently, different ingredients present in the assorted toast breads could affect greatly the fermentative production of different nucleotides by yeast. As well known, among the three isomers of inosinic acid, only 5′‐inosinic acid has flavor activity (Kuninaka, 1969). Disodium 5′‐guanylate is 3.8 times as active as disodium 5′‐inosinate although the qualitative effects of these nucleotides are virtually identical (Kuninaka, 1969).

**Table 4:** Nucleotide content in different banana‐assorted toasts^1^

Nucleotide	Content (μg/g dry matter)					
	MA15	MA 20	MA 25	MB15	MB 20	MB 25
5′‐CMP	15.4 ± 0.1^b^	16.5 ± 0.1^b^	10.4 ± 0.1^c^	16.3 ± 0.1^b^	23.5 ± 0.1^a^	88.1 ± 0.2^c^
5′‐GMP	39.6 ± 0.1^a^	40.2 ± 0.1^a^	41.0 ± 0.1^a^	35.1 ± 0.2^a^	38.6 ± 0.2^a^	33.3 ± 0.2^a^
5′‐AMP	36.2 ± 0.1^b^	34.4 ± 0.1^b^	35.0 ± 0.4^b^	35.6 ± 0.1^b^	24.8 ± 0.1^c^	39.5 ± 0.2^a^
5′‐XMP	59.0 ± 0.1^b^	57.4 ± 0.1^c^	72.0 ± 0.1^a^	35.7 ± 0.1^e^	13.0 ± 0.1^f^	39.2 ± 0.1^d^
5′‐IMP	28.4 ± 0.1^e^	16.3 ± 0.0^f^	98.7 ± 0.1^a^	42.7 ± 0.1^d^	96.4 ± 0.1^b^	65.4 ± 0.1^c^
5′‐UMP	86.6 ± 0.1^d^	147.5 ± 0.0^a^	126.4 ± 0.1^b^	36.9 ± 0.1^e^	126.6 ± 0.1^b^	103.7 ± 0.1^c^
Total	265.2 ± 0.5^e^	312.3 ± 0.3^d^	383.6 ± 0.7^a^	202.3 ± 0.3^f^	322.9 ± 0.5^c^	369.1 ± 0.7^b^

Note

*Musa sapientum* var TC2‐425 Linn [(genomically, called as *Musa* (AAA) (MA)] and *Musa basjoo* “Nam Wa” (MB). MA15, MA20, and MA25; and MB15, MB20, and MB25 indicate the percent of desiccated MA or MB powder at 15%, 20%, and 25% incorporated into the toast breads.

^1^ Data are expressed as mean ± *SD* (*n* = 3). Different superscripts in lower case in the same row indicate significantly different from each other (*p* < .05).

### 3.5 Sensory test by hedonic scoring

By 7‐point hedonic scoring (HS), we found that the top three appearance of breads were the control (HS 5.4 ± 1.2), MB15 (5.2 ± 1.3), and MB20 (5.0 ± 1.0); the color indices: the control (5.3 ± 1.2), MB15 (5.1 ± 1.1), and MB20 (4.9 ± 0.9); the flavor: MA15 (4.9 ± 1.1), MA20 (4.9 ± 1.0), and MB15 (4.8 ± 1.0) = MB25 (4.8 ± 1.1); and mouthfeel: MB20 (5.0 ± 0.9) and MA15 (4.9 ± 1.1) = MA20 (4.9 ± 1.1). The overall rank was MA15 = MB15 = MB20 = MA20 (ranking within 4.9 ~ 5.0) compared to that of control (4.5 ± 1.2; Table 5). Obviously, incorporation of an appropriate amount of the desiccated banana powder, despite MS or MB, could increase the acceptability of the customers (Table 5).

**Table 5:** The hedonic scores of different banana‐assorted toast breads^1^

	C	MA15	MA20	MA25	MB15	MB20	MB25
Appearance	5.4 ± 1.2^a^	4.8 ± 1.1^cd^	4.5 ± 1.0^d^	4.1 ± 1.3^e^	5.2 ± 1.0^ab^	5.0 ± 1.0^bc^	4.4 ± 1.2^de^
Color	5.3 ± 1.2^a^	4.6 ± 1.1^cd^	4.6 ± 1.1^cd^	4.1 ± 1.3^e^	5.1 ± 1.1^ab^	4.9 ± 0.9^bc^	4.5 ± 1.2^de^
Flavor	4.3 ± 1.2^b^	4.9 ± 1.1^a^	4.9 ± 1.0^a^	4.7 ± 1.3^ab^	4.8 ± 1.0^a^	4.7 ± 1.1^ab^	4.8 ± 1.1^a^
Mouthfeel	4.3 ± 1.3^b^	4.9 ± 1.1^a^	4.9 ± 1.1^a^	4.8 ± 1.2^a^	4.8 ± 1.2^a^	5.0 ± 0.9^a^	4.8 ± 1.2^a^
Overall	4.5 ± 1.2^c^	5.0 ± 1.0^a^	4.9 ± 1.0^a^	4.6 ± 1.3^bc^	5.0 ± 1.1^ab^	5.0 ± 1.0^a^	4.8 ± 1.1^abc^

^1^ C: control. *Musa sapientum* var TC2‐425 Linn [(genomically, called as *Musa* (AAA) (MA)] and *Musa basjoo* “Nam Wa” (MB): The amount of incorporated desiccated banana power was, respectively, 15, 20, and 25%.

### 3.6 Volatile component present in different toast breads

It was apparently seen that these two banana cultivars affected the fermentation profile differently, slightly depending, yet not well correlated in each case, on the amount of banana flour used. To name few (μg/kg, or ppm), β‐myrcene, that totally not found in MA toasts, appeared as 0.33 in 25% MB toasts. Limonene accumulated to a peak 8.88 in 20% MA compared to 20.50 in 25% MB toasts (Table 6). BHT increased to a high peak 5.79 in 25% MA toasts, but only 2.68 in 25% MB toasts. Ethanol was produced to 14.28 in 20% MA toasts, but only reached 0.84 in 25% MB toasts. Cis‐3‐nonene‐1‐ol and cis‐3‐decen‐1‐ol that did not appeared in MA fermentation were found in the MB toasts (Table 6). Obviously, the higher content of limonene, butylated hydroxytoluene (BHT), isobutyl alcohol, linalool, heptanal, and nonanal could have caused “dislike feeling” of MA25 and MB25 (Tables 5 and 6); similarly, the higher level of 3‐methyl‐1‐butanol, cis‐4‐decen‐1‐ol, and hexadecanoic acid probably could have increased the “dislike score” of the control breads (Table 6). Furthermore, higher MB content increased hardness, gumminess, and chewiness (Table 7), in addition, yielding higher acceptability (MB 15 and MB 20%, but not MB 25%) with better mouthfeel and color (Table 5).

**Table 6:** Volatile components in different banana toasts

Compounds	RI	CAS. NO	Formula	M.W.	Control	MA toast			MB toast		
						15%	20%	25%	15%	20%	25%
Terpene											
β‐myrcene	1,131	000123‐35‐3	C_10_H_16_	136	n.d.	n.d.	n.d.	n.d.	n.d.	n.d.	0.33
limonene	1,164	005989‐54‐8	C_10_H_16_	136	n.d.	0.56	8.88	5.48	8.03	5.30	20.50
γ‐terpinene	1,203	000099‐85‐4	C_10_H_16_	136	n.d.	n.d.	n.d.	0.43	0.55	0.27	1.59
o‐cymene	1,223	000527‐84‐4	C_10_H_14_	134	n.d.	0.51	0.65	n.d.	0.57	0.33	1.06
2‐carene	1,240	000554‐61‐0	C_10_H_16_	136	n.d.	n.d.	n.d.	0.11	0.11	n.d.	0.31
BHT	1897	000128‐37‐0	C_15_H_24_O	220	1.76	n.d.	0.73	5.79	2.66	n.d.	2.68
Alcohols											
Ethanol	905	000064‐17‐5	C_2_H_6_O	46	0.34	6.45	14.28	0.15	0.13	0.14	0.84
1‐propanol	1,005	000071‐23‐8	C_3_H_8_O	60	0.09	n.d.	0.66	0.11	0.18	0.14	0.24
Isobutyl alcohol	1,056	000078‐83‐1	C_4_H_10_O	74	0.44	0.42	3.84	0.20	0.64	0.97	4.20
3‐methyl‐1‐butanol	1,165	000123‐51‐3	C_5_H_12_O	88	4.68	n.d.	n.d.	n.d.	n.d.	n.d.	n.d.
1‐hexanol	1,295	000111‐27‐3	C_6_H_14_O	102	0.11	n.d.	0.90	0.30	0.18	0.15	0.95
Heptanol	1,406	000111‐70‐6	C_7_H_16_O	116	n.d.	n.d.	0.30	0.33	n.d.	n.d.	0.20
Linalool	1,493	000078‐70‐6	C_10_H_18_O	154	n.d.	n.d.	0.07	0.12	0.12	n.d.	0.19
1‐octanol	1527	000111‐87‐5	C_8_H_18_O	130	n.d.	0.82	0.23	0.18	0.12	0.08	0.23
1‐nonanol	1613	000143‐08‐8	C_9_H_20_O	144	0.29	n.d.	0.67	0.61	0.30	0.20	0.39
cis‐3‐nonen‐1‐ol	1723	010340‐23‐5	C_9_H_18_O	142	n.d.	n.d.	n.d.	n.d.	0.38	0.24	0.43
cis‐4‐decen‐1‐ol	1723	057074‐37‐0	C_10_H_20_O	156	0.55	n.d.	0.48	0.54	n.d.	n.d.	n.d.
cis‐3‐decen‐1‐ol	1733	010340‐22‐4	C_10_H_20_O	156	n.d.	n.d.	n.d.	n.d.	0.63	0.34	0.37
phenylethyl alcohol	1836	000060‐12‐8	C_8_H_10_O	122	n.d.	n.d.	n.d.	0.47	1.20	0.48	0.83
3,7‐dimethyl‐1,7‐octanediol	1576	000107‐74‐4	C_10_H_22_O_2_	174	n.d.	n.d.	0.10	0.15	n.d.	n.d.	n.d.
2,4‐decadien‐1‐ol	1918	014507‐02‐9	C_10_H_18_O	154	0.45	n.d.	0.19	0.29	0.58	0.27	0.21
2‐methyl‐1‐hexadecanol	2,338	002490‐48‐4	C_17_H_36_O	256	n.d.	n.d.	n.d.	0.24	n.d.	n.d.	n.d.
Aldehydes											
Acetaldehyde	656	000075‐07‐0	C_2_H_4_O	44	n.d.	0.01	n.d.	n.d.	n.d.	n.d.	n.d.
3‐methylbutanal	890	000590‐86‐3	C_5_H_10_O	86	n.d.	n.d.	0.71	n.d.	n.d.	n.d.	0.14
Hexanal	1,049	000066‐25‐1	C_7_H_14_O	100	n.d.	n.d.	0.24	0.07	0.07	n.d.	0.38
Heptanal	1,145	000111‐71‐7	C_8_H_16_O	114	n.d.	n.d.	0.42	0.20	n.d.	0.07	0.59
Nonanal	1,339	000124‐19‐6	C_9_H_18_O	142	0.22	n.d.	0.27	0.29	0.28	0.18	0.58
Furfural	1,393	000098‐1‐1	C_5_H_4_O_2_	96	n.d.	n.d.	n.d.	n.d.	0.08	n.d.	0.24
Benzaldehyde	1,457	000100‐52‐7	C_7_H_6_O	106	n.d.	n.d.	0.10	n.d.	0.08	n.d.	0.21
2‐nonenal	1,482	018829‐56‐6	C_9_H_16_O	140	0.23	n.d.	0.47	0.39	0.32	0.25	0.72
Benzeneacetaldehyde	1572	000122‐78‐1	C_8_H_8_O	120	n.d.	n.d.	n.d.	0.08	n.d.	n.d.	0.16
cis‐2‐decenal	1577	002497‐25‐8	C_10_H_18_O	154	n.d.	n.d.	0.08	0.19	n.d.	n.d.	n.d.
trans‐2‐decenal	1582	003913‐81‐3	C_10_H_18_O	154	n.d.	n.d.	n.d.	n.d.	0.18	0.10	0.29
trans‐2,4‐decadienal	1746	025152‐84‐5	C_10_H_16_O	152	0.66	n.d.	0.43	0.50	0.62	0.65	0.74
7‐hexadecenal	1819	056797‐40‐1	C_16_H_30_O	238	n.d.	n.d.	n.d.	n.d.	n.d.	n.d.	0.08
Hexadecanal	1983	000629‐80‐1	C_16_H_32_O	240	0.88	n.d.	n.d.	0.71	n.d.	n.d.	n.d.
Octadecanal	1986	000638‐66‐4	C_18_H_36_O	268	n.d.	n.d.	0.09	0.27	1.10	0.33	0.38
Esters											
Ethyl forrmate	798	000109‐94‐4	C_3_H_6_O_2_	74	n.d.	n.d.	n.d.	0.14	n.d.	n.d.	0.28
Ethyl acetate	855	000141‐78‐6	C_4_H_8_O_2_	88	0.12	0.23	0.07	0.40	0.23	0.11	0.88
*n*‐propyl acetate	943	000109‐60‐4	C_5_H_10_O_2_	102	n.d.	n.d.	0.20	0.09	0.16	0.10	0.07
3‐methybutyl butanoate	1,221	000106‐27‐4	C_9_H_18_O_2_	158	0.23	n.d.	0.36	0.51	n.d.	n.d.	n.d.
Isoamyl lactate	1,223	019329‐89‐6	C_8_H_16_O_3_	160	n.d.	n.d.	n.d.	0.49	n.d.	n.d.	n.d.
3‐methylbutyl isovalerate	1,252	000659‐70‐1	C_10_H_20_O_2_	172	n.d.	n.d.	0.28	0.33	n.d.	n.d.	n.d.
2,3‐butanedioldiacetate	1,314	001114‐92‐7	C_6_H_10_O_3_	174	0.11	n.d.	0.37	0.18	0.27	0.16	0.18
Methylhexyl butyrate	1,352	039026‐94‐3	C_11_H_22_O_2_	186	n.d.	n.d.	0.16	0.31	n.d.	n.d.	n.d.
Ethyl octanoate	1,382	000106‐32‐1	C_10_H_20_O_2_	172	0.63	n.d.	0.37	0.66	0.64	0.43	0.72
Isopentyl hexanoate	1,447	002198‐61‐0	C_11_H_22_O_2_	186	n.d.	n.d.	0.07	0.19	n.d.	n.d.	n.d.
Ethyl decanoate	1,590	000110‐38‐3	C_12_H_24_O_2_	200	0.76	n.d.	0.27	0.62	0.94	0.40	0.41
Ethyl dodecanoate	1816	000106‐33‐2	C_14_H_28_O_2_	228	0.60	n.d.	0.21	0.63	0.76	0.28	0.28
Ethyl tetradecanoate	1,982	000124‐06‐1	C_16_H_32_O_2_	256	0.52	n.d.	0.15	0.47	0.61	0.20	0.21
Ethyl hexadecanoate	2,107	000628‐97‐7	C_18_H_36_O_2_	284	2.95	0.10	0.93	3.00	3.16	0.97	0.95
Ethyl 9‐hexadecenoate	2,169	054546‐22‐4	C_18_H_34_O_2_	282	n.d.	n.d.	0.09	0.26	n.d.	n.d.	n.d.
Ethyl linoleate	2,502	000544‐35‐4	C_20_H_36_O_2_	308	1.45	0.23	0.32	1.12	3.53	0.93	0.86
Ethyl linolenate	969	001191‐41‐9	C_20_H_34_O_2_	306	n.d.	n.d.	n.d.	0.38	n.d.	n.d.	n.d.
Ketone											
2‐heptanone	1,142	000110‐43‐0	C_7_H_14_O	114	n.d.	n.d.	n.d.	n.d.	n.d.	n.d.	0.22
2‐nonanone	1,334	000821‐55‐6	C_9_H_18_O	142	n.d.	n.d.	n.d.	0.10	0.09	n.d.	0.25
2‐undecanone	1548	000112‐12‐9	C_11_H_22_O	170	0.27	n.d.	0.21	0.35	n.d.	n.d.	n.d.
2‐tridecanone	1,760	000593‐08‐8	C_13_H_26_O	198	0.36	n.d.	0.22	0.44	0.75	0.24	0.43
γ‐dodecalactone	1927	000706‐14‐9	C_12_H_22_O_2_	170	n.d.	n.d.	n.d.	0.16	n.d.	n.d.	n.d.
2‐pentadecanone	1934	002345‐28‐0	C_15_H_30_O	226	0.37	n.d.	0.15	0.37	0.58	0.16	0.25
Acids											
Acetic acid	1,372	000064‐19‐7	C_2_H_4_O_2_	60	1.72	1.93	0.09	2.45	3.20	2.23	2.42
Propanoic acid	1,471	000079‐9‐4	C_3_H_6_O_2_	74	0.07	n.d.	n.d.	0.11	0.21	0.10	0.13
Butanoic acid	1549	000107‐92‐6	C_4_H_8_O_2_	88	0.21	n.d.	0.93	0.55	n.d.	n.d.	n.d.
Pentanoic acid	1559	000109‐52‐4	C_5_H_10_O_2_	102	n.d.	n.d.	n.d.	0.36	0.65	0.31	0.56
Hexanoic acid	1,770	000142‐62‐1	C_6_H_12_O_2_	116	n.d.	n.d.	n.d.	0.40	0.57	0.22	0.34
Octanoic acid	1944	000124‐07‐2	C_8_H_16_O_2_	144	1.41	n.d.	n.d.	1.43	3.62	1.25	1.09
Oleic acid	2070	000112‐80‐1	C_18_H_34_O_2_	282	n.d.	n.d.	0.07	0.24	0.53	0.13	0.16
Decanoic acid	2087	000334‐48‐5	C_10_H_20_O_2_	172	3.41	n.d.	1.02	3.24	7.64	2.15	1.89
Dodecanoic acid	2,392	000143‐07‐7	C_12_H_24_O_2_	200	4.0	0.15	1.55	3.93	8.49	1.54	0.92
Tetradecanoate acid	2,582	000544‐63‐8	C_14_H_28_O_2_	228	1.86	0.91	0.81	2.12	3.01	0.67	0.86
Hexadecanoic acid	2,852	000057‐10‐3	C_16_H_32_O_2_	256	10.64	0.31	5.40	11.08	11.94	3.27	4.98
Others											
1‐ethoxy‐propane	667	000628‐32‐0	C_5_H_12_O	88	n.d.	n.d.	n.d.	n.d.	n.d.	n.d.	0.23
2‐pentyl furan	1,188	003777‐69‐3	C_9_H_14_O	138	n.d.	n.d.	n.d.	n.d.	n.d.	n.d.	0.25
3‐tert‐butyl‐4‐hydroxyanisole	2,440	000121‐00‐6	C_11_H_16_O_2_	180	4.58	0.15	0.46	4.12	6.50	3.28	2.29

Abbreviation: BHT, Butylated hydroxytoluene.

**Table 7:** The texture profile analysis of different banana‐assorted toast breads during storage

Days stored	Groups						
	C	MA15	MA20	MA25	MB15	MB20	MB25
Hardness (gw)							
0	757.1 ± 7.9^d^	917.74 ± 36.3^c^	952.1 ± 50.2^c^	1,052.8 ± 24.6^bc^	1,147.8 ± 18.5^ab^	1,136.9 ± 4.0^ab^	1,264.2 ± 176.9^a^
4	1,099.9 ± 27.0^d^	1,195.6 ± 41.7^d^	1,219.3 ± 17.7^cd^	1,350.1 ± 30.8^c^	1532.0 ± 18.8^b^	1597.9 ± 70.6^b^	1804.9 ± 182.4^a^
Springiness (gw)							
0	1.0 ± 0.0^a^	1.0 ± 0.1^a^	1.0 ± 0.1^a^	1.0 ± 0.1^a^	1.0 ± 0.1^a^	1.0 ± 0.1^a^	1.0 ± 0.1^a^
4	0.9 ± 0.0^b^	0.9 ± 0.1^ab^	0.9 ± 0.1^ab^	0.9 ± 0.1^b^	0.9 ± 0.1^b^	0.9 ± 0.1^c^	0.9 ± 0.1^b^
Cohesiveness (gw)							
0	0.7 ± 0.0^a^	0.6 ± 0.1^b^	0.6 ± 0.1^b^	0.7 ± 0.1^a^	0.6 ± 0.1^b^	0.7 ± 0.2^a^	0.6 ± 0.1^c^
4	0.5 ± 0.1^b^	0.6 ± 0.1^a^	0.6 ± 0.1^a^	0.5 ± 0.1^b^	0.5 ± 0.1^b^	0.5 ± 0.1^c^	0.5 ± 0.1^c^
Gumminess (gw)							
0	456.5 ± 7.8^c^	539.1 ± 16.3^b^	548.4 ± 41.2^b^	662.6 ± 51.5^a^	631.4 ± 91.2^a^	660.7 ± 77.5^a^	646.9 ± 90.7^ab^
4	639.1 ± 13.2^d^	700.1 ± 150.8^abc^	728.0 ± 4.0^c^	702.5 ± 40.8^ab^	803.7 ± 4.5^b^	1,145.3 ± 49.3^a^	752.2 ± 52.1^c^
Chewiness (gw)							
0	402.5 ± 4.3^c^	486.1 ± 18.7^b^	497.2 ± 39.5^b^	551.8 ± 47.2^a^	553.1 ± 84.3^a^	575.5 ± 22.1^a^	565.1 ± 81.2^a^
4	585.8 ± 9.7^d^	699.6 ± 40.5^c^	699.6 ± 5.6^c^	697.7 ± 18.3^c^	767.6 ± 153.2^ab^	826.8 ± 43.1^a^	651.1 ± 53.5^c^
Resilience (gw)							
0	0.3 ± 0.1^a^	0.3 ± 0.1^b^	0.3 ± 0.1^b^	0.3 ± 0.1^b^	0.2 ± 0.1^bc^	0.3 ± 0.1^b^	0.3 ± 0.1^b^
4	0.2 ± 0.1^a^	0.2 ± 0.1^b^	0.2 ± 0.1^b^	0.2 ± 001^b^	0.2 ± 0.1^b^	0.1 ± 0.1^b^	0.1 ± 0.1^b^

Note

Storage test was carried out for 6 days; here, only 4‐day data are presented.

As mentioned, the ethanol content reached the highest in MA20 (14.28 ppm) and the next in MA15 (6.45 ppm), compared to 0.13, 0.14, and 0.84 ppm in MB15, MB20, and MB25, as well as 0.34 ppm in the control (Table 6). The reason could be caused by an interaction function of yeast concentration, yeast activity, pH, temperature, soluble sugar content, ratio of sucrose/(glucose + fructose), and the concentration of inhibitors, if any. Thus, mathematically the overall hedonic score (HS) can be affected by factors as show:

(7) $$\emptyset \left(\text{HS}\right)=f\left(X,a,S_{i,}I_{i},\text{pH},T,t\right)$$

where *X*: the quantity of yeast cells; a: the specific activity of yeast cells; *S_i_* (*i* = 1, 2, 3,……) and *S*_1_: the starch content of strong flour; *S*_2_: the concentration of glucose; *S*_3_: the concentration of fructose; *S*_4_: the concentration of sucrose; and *I_i_* (*i* = 1, 2, 3, 4,……): the concentration of *ith* inhibitor(s). The reactor is supposed to operate at a defined pH and a constant temperature T for a duration of “*t*.”

Thus, at constant pH and T, Equation 7 reduces to

(8) $$\emptyset \left(\text{HS}\right)=f\left(A,\;\;S_{i},\;\;I_{i},\;\;t\right)$$

where *A* is the total activity of yeast cells; *A* = *aX*, others as defined in the above.

On the other hand, the concentration of acetaldehyde was “not detectable” in all breads (Table 6), implicating the downstream biochemical reactions actively undergoing to produce acetic acid, propanoic acid, pentanoic acid, ethyl formate, ethyl acetate, *n*‐propyl acetate, 2,3‐butanediol diacetate, and isopentyl hexanoate (Table 6). Interestingly, the formation of butanoic acid was only seen in the MA and the control breads (Table 6).

According to Suomalainen and Lehtonen (1978), the yeast plasma membrane regulates the movement of compounds into the yeast cells and of yeast metabolites from the cell into the medium (Suomalainen & Lehtonen, 1978). The rate of penetration of organic acids into the yeast cell depends on their lipophilic nature, their molecular size, and the degree of branching (Suomalainen & Lehtonen, 1978). During fermentation, yeast synthesizes a vast number of aroma compounds. The numerically and quantitatively largest groups of aroma compounds include fusel alcohols, fatty acids, and fatty acid esters (Suomalainen & Lehtonen, 1978). The yeast used as well as the fermentation conditions can influence the formation of aroma compounds (Suomalainen & Lehtonen, 1978). The yeast also has a profound effect on the formation of other aroma compounds, such as sulfur compounds and phenols. In addition to fermentation, the maturing process also plays an important role influencing the aroma (Suomalainen & Lehtonen, 1978). In the butyric acid production process**,** glucose must first be converted to pyruvate via the Embden–Meyerhof–Parnas pathway, which produces two moles of ATP and NADH. Subsequently, pyruvate is fermented to produce several products, depending on the strain (Dwidar, Park, Mitchell, & Sang, 2012). For acetate production, phosphotransacetylase (PTA) and acetate kinase (AK) are the key enzymes, while, for butyrate, phosphotransbutyrylase (PTB) and butyrate kinase (BK) play similar roles. During acetate production, four moles of ATP (Equation 9), while for butyrate production only three moles of ATP (Equation 10) are formed, which helps to explain why, at high growth rates, cells shift more toward acetate rather than butyrate production (Li, Han, & C. H. Zhang C.H., 2011; Michel‐Savin, Marchal, & Vandecasteele, 1990).

(9) $$C_{6}H_{12}O_{6}\to 2{\text{CH}}_{3}\text{COOH}+4H_{2}↑+2{\text{CO}}_{2}↑+4\text{ATP}$$

(10) $$C_{6}H_{12}O_{6}\to 2{\text{CH}}_{3}{\text{CH}}_{2}{\text{CH}}_{2}\text{COOH}+2H_{2}↑+2{\text{CO}}_{2}↑+3\text{ATP}$$

At the end of the exponential phase, that is, at the stationery phase, the organisms slow down acetate production and take up excreted acetate, converting it into butyrate, a mechanism of organism attempting to detoxify by reducing the total hydrogen ion concentration in the medium. Consequently, the metabolism is shifted from the more energy conserving acetate formation (Equation 9) to a lower acid content with butyrate formation (Equation 10) (Zhang, Yang, Yang, & Ma, 2009). In all MB breads, no any trace of butyric acid but relatively large amount of acetic acid was detected (Table 6).

As mentioned, galacturonic acid derived from pectin is nonfermentable and would remain in the fermented broth (Yamada et al., 1975). As MB contained higher pectin content (5.3 ± 0.3%; Table 3), while high accumulation of galacturonic acid inhibits the yeast fermentation, speculatively the ethanol production in MB should have been suppressed at a greater extent than in MS. However, interestingly, the ethanol transformation occurred more rapidly in MB (Table 6), implicating larger amount of certain yet unknown activators being present in MB rather than MS. Alternately, the role of galacturonic acid acting as an inhibitor of sugar fermentation should be considered in the design of yeast fermentation processes based on pectin‐rich feedstocks (Huisjes, Hulster, Dam, Pronk, & Maris, 1975).

Speculatively, due to having higher soluble sugar content (Table 3), MB could have driven a faster growth rate of yeast cells than MA, implicating all yeast cells were then still actively growing and maintaining in log phase with more energy conserving acetate formation.

For those strains capable of producing solvents (butanol and acetone), the fermentation usually passes through two steps—the acidogenesis phase in which butyric and acetic acids are both produced in the medium and then the solventogenesis phase in which the organism converts these acids into acetone, ethanol, and butanol (Zigova & Sturdik, 2000). This second stage is initiated as the medium becomes more acidic and the cells enter the stationary phase (Dwidar et al., 2012; Zigova & Sturdik, 2000).

The phenomena dealing with Equation 8 in fact has been previously discovered by several laboratories. Navarro and Durand (1978) indicated that during fermentation, yeast growth is rapidly halted by the increasing ethanol concentration, but fermentative activity is not entirely inhibited until high alcohol concentrations are reached (Navarro & Durand, 1978). The growth inhibitory effect of alcohol on the yeast cells is related to its retention inside within the cells, that is, when intracellular alcohol concentration reaches a maximum value (Navarro & Durand, 1978). Ethanol accumulation within the cells is a consequence of the resistance to its diffusion through the cell wall from within outside the cell (Navarro & Durand, 1978). Moreover, during fermentation with *Saccharomyces cerevisiae*, slight inhibition was noted at the 40% CO_2_ level and significant inhibition was noted above the 50% CO_2_ level, corresponding to 1.6 × 10^–2^ M of dissolved CO_2_ in the fermentor broth. High carbon dioxide content in the gas phase also inhibited the fermentation activity of baker's yeast (Chen & Gutmains, 1976). The fermentation in MA breads produced a tremendous amount of ethanol (Table 6) which definitely would be associated with huge production of carbon dioxide to trigger the “carbon dioxide inhibition.”

Moreover, the uronic acid produced by high pectin and pectinase medium can trigger the “galacturonic acid inhibition” on yeast fermentation. As the pectin content was comparable in both MA and MB (Table 3), supposedly such a type of inhibition can be neglected.

In addition, cdiGMP synthesis was lethal in the peptide background during growth on yeast nitrogen base‐glucose minimal medium (Hesketh, Vergnano, Wan, & Olivera, 2017), a similar situation like the glucose content in MA breads (13.3 ± 0.7 g/100 g; together with fructose, 12.4 ± 0.2 g/100 g = 25.7 g/100 g) (Table 3). It is thus further speculated that “Could a certain amount of 5′‐GMP present in higher concentration in the MA breads (Table 4) have been converted to a significant level of cdiGMP, resulting in suppressed growth of yeast cells?” Under such a condition, cdiGMP would also together play the role as inhibitors and change the overall activity *A*′ (Equation 8) into *A*′ (Equation 11):

(11) $$\emptyset \left(\text{HS}\right)=f\left(A^{\prime },\;\;S_{i},\;\;I_{i},\;\;t^{\prime }\right)$$

Thus in the presence of inhibitors “*I_i_*”, where *i* = 1, 2, 3, 4………; *i* = 1. 2, 3, 4………, denoting the excess ethanol (*i* = 1), carbon dioxide (*i* = 2), galacturonic acid (*i* = 3; as in above‐mentioned, this type can be negligible), and possibly cdiGMP (*i* = 4) etc. Since the content of 5′‐GMP was very comparable for MA and MB (Table 4), the contribution from cdiGMP (*i* = 4) may be also negligible. The required fermentation time thus must be corrected for “*t*” in order to obtain an optimum acceptable HA. In a defined fermentative system, the occurrence of inhibitors “*I*” (at this moment, regarding only carbon dioxide and ethanol) can be considered to be constant. And if the fermentation is operated at constant sugar level “*S,”* equation 11 reduces to.

(12) $$\emptyset \left(HS\right)=f\left(A^{\prime },\;\;t^{\prime }\right)$$

Thus, the overall hedonic scale would be simply affected by the true corrected whole activity (“*A*”) and the fermentation time “*t*” practically required:

(13) $$d\left(\Phi HS\right)=t^{\prime }dA^{\prime }+A^{\prime }dt$$

If the fermentation time “*t*” is held constant as in our case, the HS score will be only depending on the true overall microbial activity “*A*.” Speculatively, as discussed in the above, MB would have stimulated the yeast growth rate more efficiently than MA (Tables 3, 5 and 6).

### 3.7 Storage tests

#### 3.7.1 Texture profile change during storage

The hardness of all products increased during storage. No apparent difference was found for the springiness during storage for 6 days (to avoid data crowdedness, only data for 4 days are shown; Table 7). The cohesiveness declined in samples control, MA25, and all MB samples. The gumminess and chewiness all showed a tendency to increase during storage; on the contrary, the resilience reduced (Table 7). As the most acceptable sample breads were MA15, MA20, MB15, and MB20 (Table 5), it was found that the appearance and color seemed to be not the determinant factors; instead, flavor, mouthfeel (Table 5), hardness, gumminess, and chewiness (Table 7) played the key roles in deciding the acceptability (the highest HS; Table 5).

Thus, optimum recipes for manufacturing banana‐assorted toasts must be carefully handled regarding the banana species. Our study has revealed that this manufacturing technology can effectively put relief to both the banana farmers and the Government Agricultural Officers whenever the overproduction of bananas occurs.

## 4 CONCLUSION

Conclusively, the use of banana to manufacture banana‐assorted bread is an alternate acceptable way to increase the banana market value; by such a process, the banana farmers can be favored. However, different chemical constituents present in different bananas would affect the fermentative profile, that is, some recipes can be fermented at rather fast rate as evidenced by the immediate evolution of huge amount of carbon dioxide as well as ethanol. However, due to a tremendous varying constituents present in different species of bananas, the subsequent transformation of the produced huge amount of ethanol can take place very fast to form valuable flavors, while on the contrary, some will be retarded, resulting in accumulation of huge amount of ethanol in the breads, causing “disagreeable flavor and taste” of the breads. Obviously in present case, the fermentative profile has been affected by different contents of starch, soluble sugars, and pectin, whereas the acceptability (presented by the hedonic scoring) can vary as evidenced chemically by taste compounds and physically by flavor, mouthfeel, hardness, gumminess, and chewiness. Overall, the bread acceptability is affected by the fermentative profile which in turn is governed by the contents of soluble sugars, pectin, and taste compounds. Hence, regarding the use of bananas in making the banana‐assorted breads, the recipes must be carefully designed by considering such factors.

## CONFLICT OF INTEREST

The authors declare that no any conflict of interest before, during, and after this study.

## AUTHOR CONTRIBUTION

LYL, KCC, and RYP contributed to this work by designing the study, obtaining data, performing the statistical analysis, writing the manuscript, and interpreted the data. CCP and HEW participated in the conception and design of the study and acquisition of data. CSW and KHS participated in the conception and design of the study and interpretation of the data and reviewed and edited the manuscript. All authors read and approved the final manuscript.

## ETHICAL STATEMENT

The study's protocols and procedures were ethically reviewed and approved by ethical committee of Hungkuang University.

## Supporting information

Figure A1

Figure Legend
