# Supplementary figures and images for: Manufacturing technology of banana‐assorted breads: The fermentative characteristics affected by different banana cultivars
Source: Food Sci Nutr. 2020 Apr 30;8(6):2627–41. doi: 10.1002/fsn3.1539 (PMC7300050; doi:10.1002/fsn3.1539)

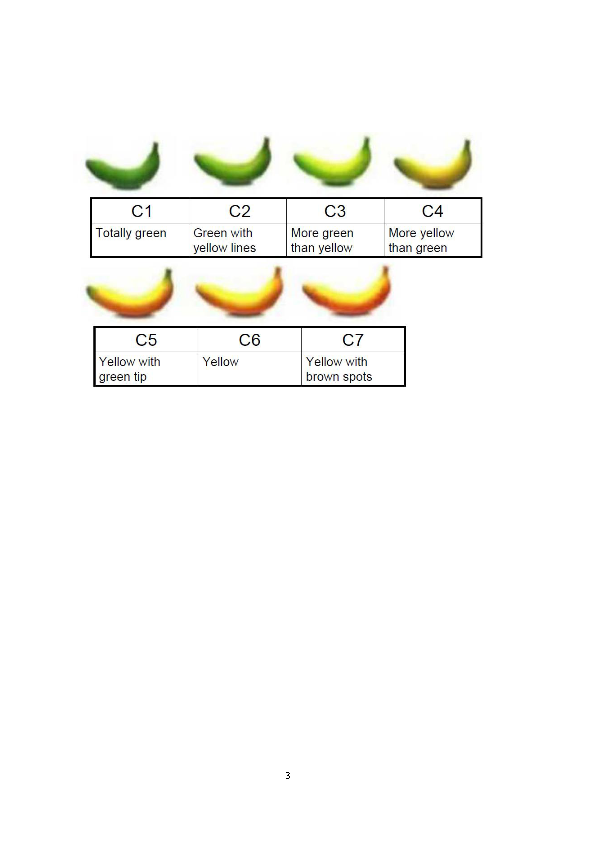

Supplement: Supplementary file 1 — Figure A1 [file FSN3-8-2627-s001.tif]
