# Supplementary material for: Manufacturing technology of banana‐assorted breads: The fermentative characteristics affected by different banana cultivars
Source: Food Sci Nutr. 2020 Apr 30;8(6):2627–41. doi: 10.1002/fsn3.1539 (PMC7300050; doi:10.1002/fsn3.1539)
Supplement: Supplementary file 2 — Figure Legend [file FSN3-8-2627-s002.docx]

**Appendix**

**Figure A1. Color index showing the degree of maturation of bananas (CEAGESP, 2006).**

The bananas color index was classified according to the commercial peel color scale (CEAGESP, 2006) as:

C1: totally green; C1: green with yellow lines; C3: more green than yellow; C4: more yellow than yellow; C5: Yellow with green tips; C6: yellow all through; and C7: yellow with brown spots.
